# Essential healthcare services provided to conflict-affected internally displaced populations in low and middle-income countries: A systematic review

**DOI:** 10.15171/hpp.2020.06

**Published:** 2020-01-28

**Authors:** Winifred Ekezie, Enemona Emmanuel Adaji, Rachael L Murray

**Affiliations:** Division of Epidemiology and Public Health, University of Nottingham, UK

**Keywords:** Primary healthcare, Early medical intervention, Armed conflict, Internally displaced persons, Low and middle-income countries

## Abstract

**Background:** Conflict and violent crises have resulted in over 40 million of internally displaced persons (IDPs). Most affected regions lack access to basic health resources and generally rely on humanitarian support. The objective of this review was to appraise primary health service interventions among conflict-induced internally displaced populations in low and middle income countries between 2000 and 2019.

**Methods:** A systematic review of literature in the following databases: Embase, MEDLINE, PsyArticles, PsycINFO, Scopus, Web of Science, LILAC and CAB Articles, was performed to identify interventions implemented in conflict IDP settings.

**Results:** Initial searches yielded 4578 papers and 30 studies met the inclusion criteria. Descriptivesynthesis analysis was used, and the final selections were assessed using a customized CriticalAppraisal Skills Programme (CASP) checklist. Included papers were from Sub-Saharan Africa, South Asia and the Middle East regions. Most studies were on prevention interventions, especially water treatment and maternal health. Treatment interventions mostly focused on onmalaria and mental health. Only one food and nutrition study with outcome data was identified, indicating limitations in IDP health-related intervention publications. Reported interventions were conducted between one week to five years, and the study qualities were moderate. The most effective interventions were integrated programmes and common challenges were weakstudy methodology and data reporting.

**Conclusion:** Regardless of the intervention types and durations, the services offered were beneficial to the IDPs. More intervention evidence are, however required as shown in gaps around food and nutrition, health education and disease surveillance.

## Introduction

Globally, there are about 41.3 million conflict and violence induced internally displaced persons (IDPs), and the majority are in low and middle-income countries (LMICs).^1^ IDPs are people forced to abandon their homes due to disasters but remain within the affected country’s borders, in contrast to refugees who migrate to other countries.^2^ IDPs, however, often experience more negative health outcomes compared to refugees.^3^ The United Nations ‘Guiding Principles on Internal Displacement’, outlines the rights of IDPs which includes protection and healthcare provision, with particular attention to women and prevention of communicable diseases.^2^ To achieve this, there are several guidelines to assist the prioritisation of interventions implemented in humanitarian conditions. These include the Sphere Handbook, World Health Organization (WHO) field manual for Communicable Disease Control in Emergency settings, and the Camp Management Toolkit.^4-6^ Nevertheless, despite being 20 years post-adoption of the internal displacement policies, many countries still struggle with the management of IDPs.^1^

Challenges experienced by displaced populations are usually health-associated and related to communicable diseases, mental health, women and children.^7,8^ Health problems faced by IDPs include increased mortality and morbidity resulting from conditions such as diarrheal diseases, measles, acute respiratory infections, malaria, and other communicable diseases which are known to account for 60%-95% of reported deaths.^9-14^ Prevalence of these conditions, most of which are vaccine-preventable, are usually highest among children^15,16^ and multiple outbreaks are also common. For example, an epidemic of dysentery can occur following a cholera outbreak.^17^

The best public health approach to managing health and well-being is health promotion and disease prevention, which are more efficient compared to treatment of existing conditions.^18^ Prevention approaches such as screening, surveillance, education, immunization and pre-disease management,^18^ are most effective in humanitarian conditions.^19-23^ These sectors fall under the WHO primary health care (PHC) and are categorized as essential healthcare services.^24^ The PHC has eight core components: disease prevention, health education; water and sanitation; food and nutrition; maternal and child health (MCH); immunization; treatment and provision of essential drugs.^24^ Each component is crucial in all humanitarian situations, which are generally characterized by excess morbidity and mortality.^14^

Despite the need for these primary healthcare services, a broader review assessing public health research evidence in humanitarian crises reported several health interventions gaps in IDP settings.^25^ The associated lack of effective interventions can also contribute to higher disease occurrence, especially communicable diseases.^11^ Hence, essential healthcare interventions in these conditions are crucial towards meeting the sustainable development goals and global health target such as the WHO vaccination coverage requirements of 90% country coverage for all national programme vaccines.^26-28^

For optimal future health care planning and management, developing interventions and effective implementation for IDPs require an understanding of the burden of diseases and previous health promotion activities.^29^ However, this is often hindered in conflict settings due to insecurity, inaccessibility of the affected areas and lack of information.^30,31^ As such, considering the increase in internal displacement from conflict and violence in the past decade,^1^ this review aims to assess the primary health interventions offered specifically to LMIC conflict-induced IDPs. The review appraises the services implemented after the adoption of the IDP policy in year 2000.^2^

## Materials and Methods

The protocol for this systematic review study was registered on PROSPERO (CRD42018086229), and the review followed the Preferred Reporting Items for Systematic Reviews and Meta-Analyses (PRISMA) guidelines.^32,33^ Before performing the study, similar reviews were searched, but none focusing on the population and locations of interest was found. Subsequently, a systematic review of published literature was performed to identify publications describing PHC interventions provided to LMIC conflict IDPs between 2000 and June 2019.

### Eligibility criteria 

Only studies conducted on populations in LMICs were considered for the review. The list of countries was adopted from the World Bank country economy classification for July 2016.^34^ Interventions of interest were the eight PHC components, as stated in the WHO alma-ata.^24^ These were education; water and sanitation; nutrition; MCH; immunization; prevention of endemic diseases; treatment; and essential drug availability. All interventions implemented at individual, group or population level were assessed.

Outcomes evaluated were intervention activities, coverage, and uptake of the services and/or resource utilization amongst IDPs. Factors affecting intervention implementation, including management approaches, stakeholder involvement, and adopted strategies, were also reviewed. All study design types with quantitative outcome data were considered. However, only published literature written in English language reporting interventions implemented from the year 2000 were included.

### Search strategy 

An initial search was conducted in January 2018 and updated in July 2019 using eight databases: CAB Articles, Embase, MEDLINE, PsyArticles, PsycINFO, Scopus, LILAC and Web of Science. The search approach was broad rather than focused on specific locations and interventions. Subject headings based on four key concepts were used for searching each database and these were (i) target population-related terms (internally displaced*or displaced*); AND (ii); conflict disaster-related terms (e.g. conflict* or complex emergency*); AND (iii) PHC intervention related terms (e.g. health*, water*, nutri* OR food*) (See Supplementary file 1).

The outcome of interest was intervention coverage after implementation of the eight PHC components. Studies specific to non-conflict IDPs were excluded, while studies with IDPs and refugees and/or asylum seekers were included only if IDP data were disaggregated. There was no fixed comparator, but comparison groups within the included studies were assessed.

Citations from the search results were imported into Mendeley reference manager for screening based on the eligibility criteria. Duplicates were removed, and assessment of titles and abstracts was conducted for the remaining publications. Full-text review was then conducted to identify IDP-focused interventions which included quantitative outcomes, before the final selection of studies. Reference lists of excluded full texts, final selected studies and related systematic reviews were examined for relevant articles.

### Data extraction and analysis

Data from all included studies were extracted into a spreadsheet in Excel. Key variables extracted included: author(s), year, country, intervention details, study population, design, setting and outcomes. Data screening and extraction were conducted independently by two reviewers and disagreements resolved through discussions with a third reviewer. Only descriptive data analysis was conducted as the studies were highly heterogeneous, and meta-analysis was not possible.

### Quality assessment

Quality assessment was conducted using a customized checklist from the Critical Appraisal Skills Programme (CASP). The customized checklist, a blend of all relevant CASP checklists, had 15 criteria and was developed to assess all study types with a single tool (see Supplementary file 2).

Each criteria was awarded one point, and the final rating was based on the proportion of positive scores. Criteria not applicable to a particular study type were perceived as ‘not applicable’ and therefore not considered in the quality calculation. The quality scores (in percentages) were ranked using three levels: weak (0-39), moderate (40-79) and strong (80-100).

## Results

### Study selection

**Figure 1:**
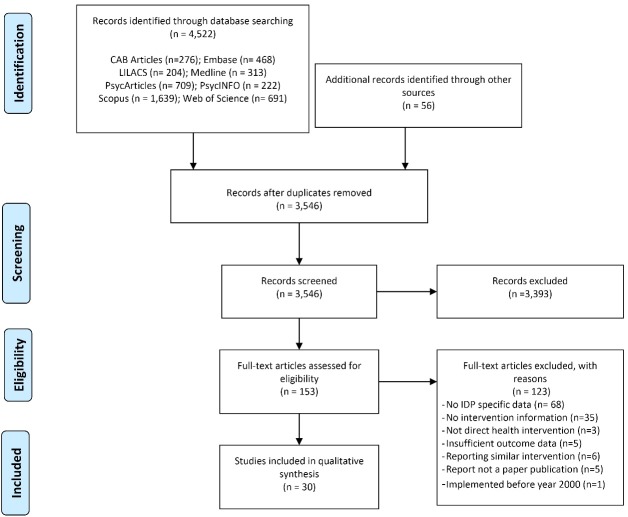
Study selection flow chart.

A total of 4578 papers were initially identified, 4522 from databases and 56 from scanning reference lists (Figure 1). After removal of duplicates, titles and abstracts of 3546 studies were screened, and 153 studies were identified as eligible for full-text review. A total of 123 studies were removed because they either had no health intervention information, reported interventions not focused on IDPs, or did not report IDP-specific data. Finally, 30 publications were included in this review (Table 1).

### Study characteristics

Most studies were conducted in six countries in Sub-Saharan Africa (22 studies), while seven studies were on four countries in South-Asia (seven studies) and one study was identified in the Middle East & North African region. Conflicts in some countries had been ongoing for over 20 years, e.g. the Democratic Republic of Congo (DRC), and some IDPs had experienced multiple displacements.^46,47^

Intervention duration ranged from one week to five years, with a median of three months. Most studies were done in camps or clinical settings used by IDPs, and sometimes included IDPs within neighbouring communities. Interventions generally targeted whole IDP populations, but a few were aimed only at women, children, or those with specific disease conditions (e.g. HIV). Most interventions were community-based (13 studies), 12 studies were at a group level, and five studies were aimed at individuals. Common study aims as reported in the studies were: to identify, assess and test intervention strategies, as well as to document experiences and strengthen health care systems.

### Essential PHC interventions

A few interventions comprised of more than one PHC component(Table 1). These included treatment (14 studies), disease prevention (11 studies), maternal and child (five studies), health education (four studies), immunization (3 studies) and food and nutrition (three studies). Some studies integrated health education when creating awareness, while essential drug interventions were considered as part of services providing medications. For this review, intervention groups were determined by the study objectives and related outcomes.

**Table 1:** Characteristics of included studies

**Author Details**	**Country**	**Intervention** ***(PHC Component) *** **Approach**	**Study population/Comparators**	**Study Design/Approach/Duration**	**Study Setting**	**Study Results**	**Quality**
Adam 2016^35^	Sudan	Reproductive health programme *(Health education, MCH)* - Emergency Reproductive Health program	Women (15–49 years)	Community pre- and post-test intervention without control - 26 months	Clinics (PHC)	Increase in women receiving home-based FP counselling (7.3% to 59.8%), current modern FP use (10.9% to 21.6%), and awareness of modern FP (60.2% to 85.0%)	Strong
Bøhler et al 2005^36^	Sudan	Tuberculosis Programme *(Treatment)* **-**National Tuberculosis Programme	Tuberculosis patients **Comparison:** Non-IDPs	Individual retrospective register analysis - 6 months	Camps and Neighbouring community	Improved treatment outcomes: cure; completed; died; failed (defaulted, transferred)]: - **New cases:** IDPs [65%; 9.3%; 4.5%; (15%, 5.7%)], Non-IDPs [43.5%; 21%; 3.6%; (21%, 9.4%)]. - **Retreatment cases:** IDPs [54.2%; 12.5%; 2.1%; (29.2%, 2.1%)], Non-IDPs [64.3%; 7.1%; 0%; (28.6%, 0%)].	Moderate
Bolton et al 2007^37^	Uganda	Mental health *(Treatment)* **-**Interpersonal psychotherapy (ITP) and creative play (CP) groups	14 to17 years old (adolescents) **Comparison :**IDPs	Group randomized controlled trial - 8 months	Camps	Reduced mean depression symptom scores: ITP (16.5%), CP (19.2%), control (17.0%)	Strong
Cunningham 2011^38^	Sudan	Reproductive Health *(MCH)* - Antenatal (ANC), postnatal (PNC) post-rape care, childbirth care and gynaecology service	Displaced women and residents	Group case study triangulation of data (documents review, interviews and observation) - Duration unclear	Non-camp populations	ANC coverage 95%; Increase in delivery uptake (3%), family planning (2%), and PNC (17%). Quality of sexually transmitted infection (STI) case management 58%.	NA
Doocy et al 2006^39^	Liberia	Water treatment *(Disease Prevention, Water & Sanitation)* - Point-of-use water treatment using flocculant–disinfectant technology	<5 children **Comparison :**IDPs	Community semi- experimental study with pre- and post-test - 3 months	Camps	Reduced diarrhoea incidence and prevalence by 90% and 83% respectively, compared with control.	Strong
Elsanousi et al 2009^40^	Sudan	Water treatment *(Disease Prevention, Water & Sanitation)* - Use of LifeStraw water filter in a tube carried around the neck.	All residents >2 years old	Community semi-experimental study with pre- and post-test 8 months	Camp	Compliance rates: Always used (86.5%), Occasionally used (9.8%) Never used (3.7%) Reduced diarrhoea incidence (15.3% to 2.3%)	Strong
Garang et al 2009^41^	Uganda	HIV Antiretroviral Therapy (ART) *(Treatment)* *-* Provision of free HIV and ART care	Adults (>=18 years) receiving ART **Comparison:** Non-IDPs	Individual intervention without pre-test 2 months	Hospital	Overall mean 4-day adherence: 99.5%. No significant difference in adherence between IDPs and non-IDPs (99.6% and 99.5%, p=0.86).	Strong
Goodrich et al 2013^42^	Kenya	HIV Care *(Essential Drugs, Treatment)* **-**Provision of HIV care	IDPs	Case study of individual medical records **-** 6 months	Clinic	23 949 patient visit compared to 23 259 previously scheduled (1.03% increase) 1420 HIV patients in IDP camps seen Basic provisions distributed to >1290 patients and their dependents.	Moderate
Hamze et al 2016^43^	DRC	Malaria *(Treatment)* - Mass drug administration; and mass screening and treatment	<5 children **Comparison:** IDPs	Group cohort intervention 3 months	Clinic	Detected 29 malaria cases through active case-finding.	Moderate
Huhn et al 2006^44^	Liberia	Yellow fever vaccination *(Disease Prevention, Immunization)* **-**Mass yellow fever immunization	Households	Community intervention without pre-test and control 3 months	Camps	Coverage rates exceeded 90% by self-report and 80% by vaccination card evidence. 97.6% IDPs informed of vaccination campaign, 91.9% vaccinated during campaign, only 83.5% had vaccination cards.	Strong
Humayun et al 2016^45^	Pakistan	Mental health *(Essential Drugs, Treatment)* - Psychosocial support and specialist care for child, treat mental disorders and offer medication.	IDP and Residents	Individual intervention without pre-test and control group - 6 months	Not clear	Drug prescription were anti-depressant (75%), anti-psychotics (10%) and anti-convulsant (8%) 60% of cases offered both pharmacological and psychological treatments.	Moderate
Jayatissa et al 2012^46^	Sri Lanka	Food distribution *(Food & Nutrition)* **-**Nutrition Rehabilitation Program offering therapeutic & supplementary food supply and blanket feeding	Children (<5 years)	Community intervention with pre- and post-test **-** 16 months	Camps	Reduction in prevalence of GAM (47%), SAM (80%) and MAM (39%). Anaemia prevalence remained high at 34% compared to national prevalence of 25%.	Moderate
Kim et al 2009^47^	DRC	HIV treatment *(MCH, Treatment)* **-** HIV and syphilis testing and HIV counselling and testing services	Women (15–49 years) **Comparison:**Non-IDP women	Random household group intervention without pre-test - 3 months	Camp and surrounding resident populations	HIV prevalence higher among IDP compared to the non-IDPs women (7.6% to 3.1%)	Strong
Kolaczinski et al 2006^48^	Uganda	Malaria treatment and management *(Essential Drugs, Treatment)* - Home-based management of fever	Caretakers of <5 children	Community intervention without pre-test and control **-** 1 week	Camps	95.0% children received correct dose and 96.3% overall adherence.	Moderate
Lee et al 2009^49^	Myanmar	Human resources for health *(Disease Prevention, Health Education, Treatment)* - Village Health Worker (VHW) partnerships malaria control	IDP-VHW and General population	Community intervention case study - 5 years	Not clear	3-fold increase in health worker density from 22 per 10 000 persons to 90 per 10 000 Increased integrated malaria control reach from 3000 to 40 000 IDPs.	Weak
Morris et al 2012^50^	Uganda	Mental health - MCH Feeding *(Food & Nutrition, MCH, Treatment)* **-**Community-based feeding program with psychosocial intervention	Mother-baby pair **Comparison**: IDP	Group semi-experimental study with pre- and post-test - 9 months	Community-based	Greater involvement with babies’ emotional responsibility and less sadness and worry among the intervention group compared to the contrast group.	Strong
Mullany et al 2010^51^	Myanmar	Maternal health care *(MCH)* - Mobile Obstetric Medics Project 3-tiered community-based maternal health workers network	Ever-married women (15–45 years)	Community-based pre- and post-test intervention without control - 3 years	General Community	Most recent pregnancy more likely to receive ANC (71.8% vs 39.3%) and other interventions. Increased PNC uptake (33.7% to 69.8%), use of modern methods to avoid pregnancy (23.9% to 45.0%) and birth taken by trained emergency obstetric care staff (5.1% to 48.7%) Reduced unmet contraception need (61.7% to 40.5%)	Strong
Nakimuli-Mpungu et al 2013^52^	Uganda	Mental health *(Treatment)* **-**Provision of routine psychological treatments including group counselling (GC)	Adults with war trauma history **Comparison**: IDP	Group quasi-experiment cohort study - 6 months	Clinic	Faster reduction in depression at 6-month and post-traumatic stress at 3-month among GC participants; Attendance to two or more sessions increased function scores Higher depression symptom scores if residing in IDP camps	Strong
Oladeji et al 2019^53^	South Sudan	Immunization *(Disease Prevention, MCH, Food & Nutrition)* - Integration of immunization into nutrition services	Children	Community and health facility intervention with pre- and post; without control - 12 months	Clinic	Increased number immunized children between 2016 and 2017: BCG (2706 vs 3411), OPV (2,449 vs 3784), Penta (2,105 vs 3700), Measles (5,680 vs 7273) Lower dropout rate in intervention than PHC centres: In Sector 2 (OR: 0.45; 95% CI: 0.36- 0.55), *P* <0.05), In sector 5 (OR: 0.27; 95% CI: 0.20 -0.35) *P* <0.05).	Moderate
Peprah et al 2016^54^	South Sudan	Cholera vaccination *(Disease Prevention, Health Education, Immunization)* - Oral cholera vaccination (OCV) campaigns and education	Adults	Individual qualitative semi-structured interviews without control - 3 months	Camps	OVC reached 85–96% of the target population. Heightened fear of disease and political danger contributed to camp residents’ perception of cholera More trust in the United Nations and NGO staff providing vaccine not the national government	Strong
Pinto et al 2005^55^	Sudan	Surveillance *(Disease Prevention)* - Early Warning System using national communicable disease surveillance system	IDP Camps	Community intervention without pre-test and control - 3 months	Camps	76% of camps reported data regularly after 10 weeks of implementation 179,795 consultations reported included ARI (18.7%), malaria (15%), bloody diarrhoea (8.4%); and SAM (1%). More than 1,000 cases of acute watery diarrhoea reported. Two outbreaks of Shigella dysenteriae detected 868 deaths reported	Moderate
Richards et al 2009^56^	Myanmar	Malaria control *(Disease Prevention, Health Education, Treatment)* **-**Integrated malaria control	All IDPs	Community intervention with pre- and post-test - 27 months	Clinics	Reduction in *P. falciparum* prevalence (8.4% to 1.1%); annual incidence (232 to 70 per cases/1000/year) Improvement in household members sleeping under an LLITN (0% to 89%) and malaria knowledge in all areas. Higher mean number of IDPs per net owned compared to non-IDPs (3.1 vs 2.7)	Moderate
Sami et al 2017^57^	South Sudan	Newborn health services *(MCH)* - Facility-based newborn health services	Mothers	Clinical observation and qualitative interview without pre-test and control - 3 months	Camp clinics	Minimal time is spent on PNC by staff (6.2%), PNC less consistently monitored (27.7%) and deliveries by skilled attendants more likely to receive PNC monitoring Selected components commonly practised: thermal care (62.5%), infection prevention (74.8%), and feeding support (63.6%) Poor availability of essential drugs compared to requirements at primary care level (9 of 25) and hospital (20 of 37) Poor mothers’ knowledge of danger signs: fever (44.8%), not feeding well (41.0%), difficulty breathing (28.9%), reduced activity (27.7%), feeling cold (18.0%) and convulsions (11.2%).	Strong
Sonderegger et al 2011^58^	Uganda	Mental health *(Treatment)* **-**Culturally sensitive and cognitive behaviour therapy	War-affected IDPs **Comparison**: IDPs	Group randomized intervention with pre- and post-test - 1 month	Camps	No significant differences between groups at pre-assessment, but significant differences found at post-assessment and 3-month follow-up. Treatment group had lower scores on the depression-like syndromes and the anxiety-like syndrome; and more prosocial behaviours.	Moderate
Spencer et al 2004^59^	Uganda	Malaria prevention *(Disease Prevention)* - Mass distribution of ITNs	All IDPs	Community intervention with pre- and post-test - 1 month	Camps	75.6% households had ITNs, but only 56.5% slept under ITNs Prevalence of malaria parasitaemia (11.2%) significantly lower in ITN users compared to non-users (9.2% vs 3.8%) Fever higher in <5 years by 7.2%	Strong
Steele et al 2008^60^	Uganda	Water treatment *(Water & Sanitation)* *-* Disinfection of jerry cans using high strength sodium hypochlorite	Households **Comparison**: IDPs	Group intervention with pre- and post-test - 1 month	Camps	Jerry can contamination not come from water source. Source consistently tested 0cfu/100ml microbiological contamination, but jerry can could not reduce to 0 cfu/100 mL Chlorine strength depleted after repetitive cleaning	Moderate
Walden et al 2005^61^	Sudan	Water treatment *(Disease Prevention, Water & Sanitation)* **-**Mass disinfection of water containers	All IDPs	Community intervention without pre-test - 3 months	Camp	Reduction in diarrhoea cases after cleaning campaign. 88% of containers in the camp disinfected Random residual chlorine in 172 containers showed chlorine level of about, 0.22 mg/L	Weak
Watson et al 2019^62^	Iraq	Handwashing *(Water & Sanitation)* - Toy-in-soap intervention	Children **Comparison**: IDPs	Group randomized controlled intervention with pre- and post-test - 1 month	Camp	Baseline intervention vs control (24% vs 32%) Endline intervention vs control (40% vs 13%) Intervention 4 times more likely to handwash (adjusted RR=3.94, 95% CI: 1.59–9.79)	Strong
Wayte et al 2008^63^	Timor-Leste	Sexual and Reproductive Health *(MCH)* - Comprehensive Reproductive Health Strategy with key focus on Safe Motherhood.	General IDP review	Hospital-based intervention without pre-test - 6 months	Community	Mobile ANC services provided to 29 of 56 camps but ceased after two months. 16 of 399 women seen for ANC during mobile clinics Two dozen tents on the grounds of national hospital to improve hospital-based births and emergency obstetric care 261 pregnant women transferred	Moderate
Zhou et al 2016^64^	Myanmar	Malaria control *(Disease Prevention, Treatment)* - Malaria and vector surveillance	All IDP **Comparison**: Non-IDP	Community intervention without pre-test - 40 months	Camps/ Community Clinics	Annual clinical malaria incidence rates lower among IDPs compared to non-IDPs (38.8 vs 127.0 cases/1000 person-year) Seasonal malaria outbreaks unchanged in local villages but increased about 10-fold in IDP camps Over 99% of households in both communities owned bed nets, but more IDPs used LLITNs (60.9% vs 0.4%).	Strong

*Note:* Antenatal care (ANC), Antiretroviral Therapy (ART), Confidence Interval (CI), Family Planning (FP), Global-, Severe- and Moderate- Acute Malnutrition (GAM, SAM, MAM), Human Immunodeficiency Virus (HIV), Internally Displaced Person (IDP), Long Lasting Insecticide Treated Net (LL- ITN), Maternal and Child Health (MCH), Primary Health Care (PHC), Postnatal care (PNC), Relative Risk (RR).

### Intervention outcomes

The results were grouped under four areas: disease prevention, food and nutrition; sexual, reproductive, MCH; and treatment and essential drugs. Observed outcomes showed mostly positive changes and improvement after implementation of the interventions. To avoid duplication, malaria interventions which included both preventive and treatment measures were presented only within the treatment section.

### Disease prevention interventions

Intervention studies described in the disease prevention category were those related to water, sanitation, vaccination, and surveillance. Water treatment interventions mostly aimed to reduce diarrhoea incidence by minimizing contamination, by disinfection of either point-of-use^39^ or water storage,^40,60,61^ and both approaches were considered effective. Steele et al^60^ observed that water contamination did not come from water sources but storage vessels. The study by Doocy et al^39^ supported this when no difference was observed in water recontamination between comparator groups (water treatment vs no treatment) after the provision of improved storage vessels. However, treatment of water storage lost strength overtime so required occasional retreatment.^60,61^ Hence, although storage treatments showed immediate results, continuous disinfection was considered intensive and expensive.^40,61^ Intervention compliance rates were very high, especially when the services were offered free-of-charge.^40^ Overall, water treatment interventions showed a reduction in diarrhoea incidence by about 90%^47^ and prevalence reduction by over 80%.^39,40,61^

With respect to sanitation, Watson et al^62^ presented the result of an incentive-based hygiene promotion intervention targeted at children. The intervention encouraged handwashing among children between the ages of five and 12 through play and curiosity motivation; and showed children incentivised with a toy embedded within soaps were four times more likely to hand wash compared to those offered only soaps (relative risk, RR=3.94, 95%; confidence interval, CI: 1.59–9.79). This study outcome highlighted the impact of incentives on intervention uptake.

The vaccination study by Oladeji et al^53^ which evaluated the effect of integrating immunization services within nutrition services reported improvements in vaccination uptake and dose completion. The study showed that children in the two selected sites offering a combination of immunization and therapeutic food were about 27% and 45% less likely to miss vaccination compared to those vaccinated at primary healthcare centers with no food program (odds ratio, OR: 0.45; 95% CI:0.36-0.55, *P* < 0.05) and (OR: 0.27; 95% CI: 0.20 -0.35, *P* < 0.05) respectively.^53^ The other two vaccination studies focused on specific diseases and aims: yellow fever vaccination administration and effectiveness^44^ and IDP perception of cholera vaccination.^54^ Both studies reported exceeding the 90% WHO vaccination coverage standard requirement; and the high coverage level was linked to the IDPs’ high of level awareness about the disease severity.^54^ These coverage assessments were however based on self-reports since most IDPs had no vaccination cards to present as evidence.^44^ There were observed slight reductions in the number of IDPs vaccinated compared to the total numbers initially informed of the vaccination schedule.^44,54^ Factors that contributed to the reduction and vaccination refusal were the presumed side effects, political influence and fear the vaccines would be used as a weapon against them.^54^ Consequently, some IDPs trusted non-governmental organizations to provide them with vaccination rather than the national government.^54^

Outputs from the surveillance study showed that when an early warning system was used in camps, disease incidence and mortality were identified and reported quicker.^55^ A major benefit from the surveillance system was the increased ability to detect outbreaks of uncommon diseases such as hepatitis E more quickly.

### Food and nutrition interventions

Although three studies had a food and nutrition component,^46,50,53^ only one reported related outcome data on the intervention uptake.^46^ The study by Jayatissa et al^46^ showed reduced levels of all forms of acute malnutrition [Global-, Severe- and Moderate- Acute Malnutrition (GAM, SAM, MAM)] between 39% and 80%. The study also monitored for anaemia (another potential indication of nutritional deficiency) and observed that although there was a reduction in malnutrition, anaemia levels remained higher than the national prevalence at 34% compared to 25%. This difference highlights the need for further research to investigate this finding.

### Sexual, reproductive, maternal and child health interventions

Family planning (FP) interventions all showed increased service uptake. One study on the association between home-based counselling and use of FP reported that the proportion of women receiving home-based FP counselling increased from 7.3% to 59.8%, and those aware of modern FP methods increased by over 20%.^35^ Wayte et al,^63^ however, reported fewer women received FP and antenatal care (ANC) in camps, yet ANC uptake was higher compared to other MCH services. Also, obstetric care provided in the study by Mullany et al^51^ showed significant uptake, especially among women with recent pregnancies. Poor uptake of some services could be explained in the study implemented in a non-camp setting which showed IDP women expressed poor privacy satisfaction (63%) and inadequate provision of required needs.^38^ Sexually transmitted diseases (STIs) and HIV/AIDS were not a major focus in the SRH interventions.^35,38,51,57,63^ Most interventions lasted for longer durations (up to three years)^35,51^ compared to other intervention types. Reasons given for shorter SRH intervention periods included poor sustainability and high-cost implications.^63^

MCH intervention improvements were attributed mainly to the increased availability of healthcare resources, i.e. health workers and essential supplies, especially the provision of FP commodities for pregnancy risk reduction.^51,57^ A study on newborn care reported lack of newborn services and wide disparities in the care provided at primary care levels compared to secondary care (hospitals). Difference in the quality of health services between the two levels of care was evident in the higher number of recommended essential drugs available at the hospitals (20/37) compared to primary care facilities (9/25).^57^ The observed service differences also influenced health outcomes. For example, compared to the hospitals, the relative risk of infection 1.28 (CI: 1.11-1.47), indicated a 28% higher risk of getting an infection at the primary care. Nevertheless, both studies showed that mothers whose births were conducted by skilled attendants were more likely to take up postnatal care (PNC). Observations in the same study also showed poor knowledge of newborn danger signs among new mothers, and that midwives spent more time on non-patient activities due to staff shortage.^57^ Largely, the availability of MCH services at clinics created positive awareness among mothers and increased usage of health facilities.

### Treatment and essential drugs interventions

Studies on treatment and essential drugs focused on specific disease conditions, including tuberculosis (TB), HIV and malaria. However, not all studies targeting specific diseases included a treatment component or offered drugs/medications, e.g. studies on diarrhoea.

Bøhler et al^36^ compared TB treatment uptake between IDPs and non-IDPs and observed treatment coverages of 65% and 54.2% cure for new and retreatment of IDP cases respectively, and 43.5% and 64.3% for non-IDPs were unsatisfactory when compared to the WHO TB treatment target of 85%.^65^ However, outcomes for retreatment cases was reversed with non-IDPs having higher cure rates (54.2% to 64.3%). Hence, TB treatment completion rates were lower among IDP new cases but higher for retreatment cases. The conflicting observations were linked to IDP migration, which caused a break in the treatment period and resulted in more IDPs needing retreatment.

All HIV studies focused on HIV management and adherence.^41,42,47^ Adherence to treatment was generally high, with one study reporting mean adherence of 99.6% and no significant difference when compared to non-IDPs.^41^ Key factors that influenced non-adherence among IDPs were being at the first stages of treatment and condemning attitudes of health staff.^41^ The study by Kim et al^47^ showed significant associations between recent STI symptoms and conflict-related sexual violence among IDP women compared to non-IDPs (aOR=3.9 and 4.2; *P* value <0.01 and =0.05, respectively). This indicated HIV care was of high priority in conflict settings and possible if planned effectively as shown in the study by Goodrich et al^42^ which documented the experiences of offering HIV care in active conflict settings. Goodrich et al^42^ advised for the inclusion of rapid case tracking, availability of resources, staff support and promotion of patient and community networks in HIV programs. Findings from the TB and HIV studies illustrated the impact of conflict and migration on treatment uptake.

Malaria prevention using insecticide-treated nets (ITNs) showed high uptake among the IDPs.^56,59^ Spencer et al^59^ reported ITN availability in about 75.6% of households, but only 56.5% of IDPs slept under them, and most often the nets were damaged. Despite that, net usage was observed to be a protective factor, as shown by the higher malaria prevalence among non-ITN users. The study also monitored haemoglobin concentration and discovered no difference between users and non-users, but observed fever was highest among children <5 years.^59^ A study on community-based integrated malaria control programme which offered malaria education reported increased use of LLITNs from 0% to 89%.^56^

For malaria-infected individuals, active case finding was reported as the best approach to identifying cases for treatment.^43^ The study which compared active case finding approach between IDPs and non-IDPs discovered more humanitarian aid intervention support was offered to IDPs compared to non-IDPs.^64^ The increased support was stated to have likely contributed to the higher rates of bed net usage among IDPs compared non-IDPs^43^; and also to the lower annual clinical malaria incidence in IDP camps compared to surrounding villages.^64^ However, the overall incidence was still higher among IDPs, compared to non-IDPs in the other study. Richards et al^56^ found similar results with the mean number of IDPs owning a net being higher compared to resident villagers (3.1 to 2.7). Active case finding study comparison between IDP groups showed that in households where one person was infected with malaria, other members were not more likely to be infected.^43^ This discovery implied that household malaria screening interventions were not effective, and malaria was not contagious. However, malaria incidence was still greater among IDPs compared to non-IDPs in other studies.^64^ High malaria prevalence, since not contagious, was attributed to the high vector population observed in IDP camps compared to non-IDP areas.^64^ In addition, seasonal variation of malaria influenced prevalence rates and led to outbreaks, among both IDPs and non-IDPs.^64^ Results from the studies suggested the most cost-effective malaria control strategy was screening of symptomatic patients (symptom-based screening). But the most comprehensive approaches advised were mass drug administration and mass screening and treatment.^43^ Regardless of intervention methods, the malaria studies showed that for any strategy to be effective, it was fundamental to involve multiple groups such as caretakers, community drug distributors and community/villages health workers.^43,48,49^ Malaria treatment side effects were also observed in some cases,^48^ but proper education was identified to be effective in encouraging proper usage and adherence.^59^

There were more studies conducted on mental health compared to any other health conditions. The most commonly studied mental health conditions were anxiety and depression, while and the main intervention technique used was group activity therapy. The individual-level study by Humayun et al^45^ which compared outcomes with non-IDPs reported most IDP mental health symptoms existed before displacement (60%), with only 18.7% occurring after displacement. Studies on depression, anxiety, and PTSD reported reductions and an overall improvement in mental function among intervention groups compared to control groups.^37,50,52,58^ Improvements were often not observable at the early stages of the interventions, for example within the first three months. However, even minimal engagement within the intervention proved beneficial compared to no intervention at all.^37,52,58^ Nakimuli-Mpungu et al^52^ observed higher depression symptom scores among IDPs living in camps (OR=2.40, CI: 1.10-5.23), implying IDP camp residents were about two times more likely to experience depression. A group therapy study which incorporated mental health and MCH components observed that reduction of worry and sadness among mothers increased their involvement in childcare and feeding, and this helped reduce child malnourishment.^45^ Only one study reported an integrative intervention of pharmacological and psychological treatments, and this combination was offered to about 60% of cases.^45^ Overall, education provided on anxiety and coping skills were effective in relieving symptoms of mental health, and group therapy approaches were considered the most acceptable and cost-effective among the displaced populations.

## Discussion

This review aimed to assess the primary health interventions offered to IDPs in conflict regions. Thirty published studies were identified on varied intervention programs implemented since the year 2000, and all these interventions lasted for less than five years. The included papers described the different strategies adopted in humanitarian settings; however, representation of the PHC components was uneven. This suggested that although internal displacement occurs for prolonged periods (decades), most interventions do not last as long. It also showed only a small number of studies had reported health interventions in conflict displacement settings through research publications. This finding highlights health intervention evidence gaps in conflict IDP settings, thus, supporting the observations from other reviews.^25,66^

Conflict-induced displacements are most times complex and hamper humanitarian efforts,^12,67^ and from the included studies, factors observed to influence intervention implementation included security, politics, IDP migration and climate seasonal changes. Recommendations by the authors to address the shortage and uneven distribution of interventions included the need for more integrative intervention studies, increased health education and extended research duration to allow for proper monitoring of long-term intervention effects. Quality of the included studies was moderately strong considering challenges faced in conducting research in conflict regions, such as ethical limitations related to feasibility, beneficence and human rights issues of doing research in such settings.^68,69^ The main study quality weaknesses were lack of clear study criteria, limited information on bias, poor data stratification and weak generalizability potential.

### Health intervention effectiveness

Interventions offering multiple PHC components appeared to have increased uptake and coverage the most.^53,70^ As such, although water treatment and sanitation improvement studies highlighted the effectiveness of each component, combination of both as recommended by the Sphere guidelines,^6^ would have offered more efficient and beneficial outcomes.

In contrast to findings from other reviews with focus on boarder humanitarian settings and scope,^25,66^ only one food and nutrition study met the criteria for this review. This highlighted the gap in interventions related to conflict-induced IDPs. The one included food and nutrition study used the common humanitarian food distribution approach, which is rationing.^23^ Although, composition of the food supplements provided did not meet the nutritional need of the target population.^46^ Rationing is considered an effective method of ensuring whole populations have access to resources offered; however, for food and nutrition interventions, this often requires micronutrient standardization to ensure individual nutritional needs are met.^23^ The review by Balhara et al^66^ on the impact of nutrition interventions further outlined other effective food-related interventions; these included ready-to-use therapeutic foods, micronutrient supplementation, and food and cash transfers. Integration of these different options based on specific target groups could improve nutritional outcomes.^71,72^

Surveillance and immunization studies were limited but significantly effective where implemented. Thus, previous reviews have highlighted the importance of research in both areas in humanitarian settings.^30,73,74^ The study on IDP perceptions to immunization revealed valuable insights on vaccination, including fear of vaccines being used as a weapon against them. ^54^ Awareness of such belief can inform appropriate vaccine communication during intervention development.^75^

Although mental health services are not commonly managed at PHC levels, it is considered a critical intervention in humanitarian conflict conditions.^76^ Mental health interventions were the most multi-focused in terms of outcomes of interest and study tools used. Also, this approach follows the WHO recommendation to integrate mental health services into other health interventions in emergency settings.^77^

Overall, the most effective interventions were those that targeted and reached more IDPs, and this included the provision of healthcare workers, population-level distribution of resources and disease surveillance. Achieving optimal intervention delivery, therefore, requires innovative strategies which include program combinations, use of pre-existing structures and focus on health system strengthening.^36,45,46,55,63^

### Factors influencing health interventions

The review showed that the use of already existing structures amplified intervention successes and reduced costs.^49,53,55^ Key contributors to high intervention uptake among the IDPs were availability of incentives, free services (e.g. water treatment, ITNs), and personal awareness of either general health conditions (e.g. FP) or specific disease severity (e.g. vaccine-preventable diseases). For treatment interventions, disease stages also contributed to intervention complexity. For example, adherence and cure of chronic diseases such as TB and HIV required a certain level of consistency, which cannot be predictable for displaced populations. Impact of these limitations corroborates with other studies and intervention types.^73,78-80^

Consideration of community characteristic of affected populations is also essential when providing health interventions. One study showed that community characteristics in conflict regions, especially education and poverty, were more associated with the uptake of maternal healthcare services than individual characteristics.^81^ However, similar evidence was lacking in most of the studies reviewed. Inclusion of more community-level information and health education interventions would likely improve intervention uptake and effectiveness.

### Study limitations

Wide variations in study aims, intervention approach and countries reported made it difficult to perform extensive comparisons. The narrowed inclusion criteria with a focus on quantitative data may have led to the exclusion of some relevant peer-reviewed literature. Also, the use of only English language articles probably introduced some publication bias. As most studies were interventions with no pre-test and comparison groups, attribution and changes specifically related to the intervention could not be adequately assessed. Also, grey literature which may have had relevant information and interesting information on issues with executing intervention studies in conflict settings, such as specific ethical concerns, were not included in this review. Identified difficulties in conducting research in environments of significant need, such as humanitarian crisis settings, likely limited the volume of peer-review publications. Nevertheless, the findings in this review were similar to observations in related studies and highlight the fact more reviewed publications need to be encouraged so as to increase emphasis on implementation science.

## Conclusion

This review demonstrated a variety of intervention programs could be implemented among IDPs in conflict settings and, irrespective of the program package; these can be effective and beneficial to IDPs. However, the evidence base is minimal, and several challenges – including security concerns in conflict regions and ethical implications, were identified to affect the ability to carry out rigorous studies among such populations who are in significant need. Nevertheless, documentation of past intervention experiences is strongly encouraged to support future development strategies and ensure more cost-effective interventions are designed for conflict disaster settings. In addition to research on delivering of interventions, more peer-reviewed publications reporting conflict displacement interventions with outcome data would be beneficial for identifying effective and efficient best practices as well as increase the quality of research in the humanitarian sector.

## Ethical approval

Not applicable.

## Competing interests

None to declare.

## Funding

This research received no grant from any funding agency or organization.

## Authors’ contributions

WE and RLM designed the research and conducted the systematic research. WE and EEA extracted the data and conducted study quality assessments. WE analysed the data and wrote the manuscript. All authors read and approved the final manuscript.

## Disclaimer

This report presents independent research conducted by the authors. The views and opinions expressed by authors in this publication are those of the authors and do not necessarily reflect those of the University of Nottigham.

## Acknowledgments

This manuscript was part of a PhD research and we would like to thank Stephen Timmons, Puja Myles, Catherine Pritchard, Penelope Siebert and Manpreet Bains for their contribution to the supervision process.

## Supplementary Materials

Supplementary file 1. Sample search strategy from EMBASE Database.

Supplementary file 2. Customized CASP Study Quality Checklist.
