# Supplementary material for: Essential healthcare services provided to conflict-affected internally displaced populations in low and middle-income countries: A systematic review
Source: Health Promot Perspect. 2020 Jan 28;10(1):24–37. doi: 10.15171/hpp.2020.06 (PMC7036202; doi:10.15171/hpp.2020.06)
Supplement: Supplementary file 1 [file hpp-10-24-s001.pdf]

Supplementary file 1. Sample search strategy from EMBASE Database

# ▲	Searches	Results	Type
1	(internally displaced* or displaced*).mp. [mp=title, abstract, heading word, drug trade name, original title, device manufacturer, drug manufacturer, device trade name, keyword, floating subheading word, candidate term word]	37137	Advanced
2	limit 1 to (human and yr="2000 -Current")	16507	Advanced
3	(conflict* or complex emergency*).mp. [mp=title, abstract, heading word, drug trade name, original title, device manufacturer, drug manufacturer, device trade name, keyword, floating subheading word, candidate term word]	165704	Advanced
4	1 and 2 and 3	540	Advanced
5	health*.mp. [mp=title, abstract, heading word, drug trade name, original title, device manufacturer, drug manufacturer, device trade name, keyword, floating subheading word, candidate term word]	4836123	Advanced
6	(water* or sanitation*).mp. [mp=title, abstract, heading word, drug trade name, original title, device manufacturer, drug manufacturer, device trade name, keyword, floating subheading word, candidate term word]	1104238	Advanced
7	(nutrit* or food*).mp. [mp=title, abstract, heading word, drug trade name, original title, device manufacturer, drug manufacturer, device trade name, keyword, floating subheading word, candidate term word]	1159647	Advanced
8	(vaccin* or immuni*).mp. [mp=title, abstract, heading word, drug trade name, original title, device manufacturer, drug manufacturer, device trade name, keyword, floating subheading word, candidate term word]	839762	Advanced
9	prevent*.mp. [mp=title, abstract, heading word, drug trade name, original title, device manufacturer, drug manufacturer, device trade name, keyword, floating subheading word, candidate term word]	2646083	Advanced
10	educat*.mp. [mp=title, abstract, heading word, drug trade name, original title, device manufacturer, drug manufacturer, device trade name, keyword, floating subheading word, candidate term word]	1245171	Advanced
11	treat*.mp. [mp=title, abstract, heading word, drug trade name, original title, device manufacturer, drug manufacturer, device trade name, keyword, floating subheading word, candidate term word]	7802736	Advanced
12	(drug* or medic*).mp. [mp=title, abstract, heading word, drug trade name, original title, device manufacturer, drug manufacturer, device trade name, keyword, floating subheading word, candidate term word]	13494116	Advanced
13	4 and 5	369	Advanced
14	4 and 6	30	Advanced
15	4 and 7	72	Advanced
16	4 and 8	35	Advanced
17	4 and 9	101	Advanced
18	4 and 10	70	Advanced
19	4 and 11	139	Advanced
20	4 and 12	169	Advanced
21	13 or 14 or 15 or 16 or 17 or 18 or 19 or 20	468	Advanced
