# Supplementary material for: Essential healthcare services provided to conflict-affected internally displaced populations in low and middle-income countries: A systematic review
Source: Health Promot Perspect. 2020 Jan 28;10(1):24–37. doi: 10.15171/hpp.2020.06 (PMC7036202; doi:10.15171/hpp.2020.06)
Supplement: Supplementary file 2 [file hpp-10-24-s002.pdf]

Supplementary File 2. Customized CASP Study Quality Checklist

Study No		Author Details	
Study Title			
Study Design		Is there a clear study result?	
Is there a clear research focus?		Are ethical issues and limitations reported?	
Is an appropriate methodology used?		Are result applicable and replicable?	
Is appropriate recruitment done (with stats)?		Does it fit with other related evidence?	
Is there a comparison group?		What are the study Implications and/or Recommendations?	
Is bias minimized with clear inclusion and exclusion criteria?		Quality Summary Note	
Are confounders accounted?		Quality Score	
List of accounted confounders		Quality Grade	
Are comparison groups similar?			
Is follow up completion rate reported?			
Are loss to follow up reasons reported?			
